# Alter between gut bacteria and blood metabolites and the anti-tumor effects of *Faecalibacterium prausnitzii* in breast cancer

**DOI:** 10.1186/s12866-020-01739-1

**Published:** 2020-04-09

**Authors:** Ji Ma, Lingqi Sun, Ying Liu, Hui Ren, Yali Shen, Feng Bi, Tao Zhang, Xin Wang

**Affiliations:** 1 Department of Medical Oncology and Laboratory of Molecular Targeted Therapy in Oncology, State Key Laboratory of Biotherapy, West China Hospital, Sichuan University, Chengdu, Sichuan Province, 610041 PR China; 2 Southwest medical university, Luzhou, Sichuan Province 646000 PR China; 3 Department of Neurology, The Air Force Hospital of Western Theater Command, Chengdu, Sichuan Province 610041 PR China; 4 Departments of Breast Surgery, The 940th Hospital of Joint Logistics Support Force of Chinese People’s Liberation Army, Lanzhou, Gansu Province 730000 PR China; 5 Department of Breast Surgery, Fudan University Cancer Hospital, Shanghai, 200000 PR China; 6 Department of Oncology, The General Hospital of Western Theater Command, Chengdu, Sichuan Province 610041 PR China

**Keywords:** Breast cancer, Gut bacteria, Blood metabolites, *Faecalibacterium prausnitzii*, IL-6; STAT3

## Abstract

**Background:**

The aim was to evaluate the changes of 16S rDNA sequencing and LC-MS metabolomics in breast cancer and explore the growth inhibition of breast cancer cells by *Faecalibacterium prausnitzii*.

**Results:**

Total 49 significantly different flora and 26 different metabolites were screened between two groups, and the correlation was calculated. Relative abudance of *Firmicutes* and *Bacteroidetes* were decreased, while relative abundance of *verrucomicrobla*, *proteobacteria* and *actinobacteria* was increased in breast cancer group. Differentially expressed metabolites were mainly enriched in pathways such as linoleic acid metabolism, retrograde endocannabinoid signaling, biosynthesis of unsaturated fatty acids, choline metabolism in cancer and arachidonic acid metabolism. Lipid upregulation was found in breast cancer patients, especially phosphorocholine. The abundance of *Faecalibacterium* was reduced in breast cancer patients, which was negatively correlated with various phosphorylcholines. Moreover, *Faecalibacterium prausnitzii*, the most well-known species in *Faecalibacterium* genus, could inhibit the secretion of interleukin-6 (IL-6) and the phosphorylation of Janus kinases 2 (JAK2)/signal transducers and activators of transcription 3 (STAT3) in breast cancer cells. *Faecalibacterium prausnitzii* also suppressed the proliferation and invasion and promoted the apoptosis of breast cancer cells, while these effects disappeared after adding recombinant human IL-6.

**Conclusions:**

Flora-metabolites combined with the flora-bacteria (such as *Faecalibacterium* combined with phosphorocholine) might a new detection method for breast cancer. *Faecalibacterium* may be helpful for prevention of breast cancer. *Faecalibacterium prausnitzii* suppresses the growth of breast cancer cells through inhibition of IL-6/STAT3 pathway.

## Background

Breast cancer is a malignant tumor that occurs in the epithelial tissue of the breast gland [1]. The breast is not an important organ for maintaining human life and in situ breast cancer is not fatal [2]. However, since breast cancer cells lose the characteristics of normal cells, the connections between the cells are loose and easily fall off [3]. Once the cancer cells fall off, the free cancer cells can spread with the blood or lymph, forming a metastasis and endangering life. Breast cancer has become a common tumor that threatens women’s physical and mental health [4]. Early detection and early diagnosis of breast cancer is the key to improve the efficacy [5]. The diagnosis and differential diagnosis of breast cancer should be performed in combination with the patient’s clinical manifestations and medical history, physical examination, imaging examination, histopathology and cytopathology [6]. With the deepening understanding of the biological behavior of breast cancer, as well as the transformation and renewal of the treatment concept, the treatment of breast cancer has entered the era of comprehensive treatment, forming a treatment mode of both local and systemic treatment of breast cancer [7]. According to the stage of the tumor and the physical condition of the patient, the doctor will use surgery, radiotherapy, chemotherapy, endocrine therapy, biological targeted therapy and Chinese medicine adjuvant therapy as appropriate [8]. However, the prognosis of breast cancer is not satisfactory. In order to improve breast cancer screening and provide more therapeutic targets, new technologies and protocols are urgently needed to study the deeper mechanisms of action of breast cancer.

16S rDNA refers to the DNA sequence corresponding to the ribosomal 16S rDNA molecule in the bacterial genome, which is the coding gene of 16S rDNA [9]. The 16S rDNA sequence consists of 10 Conserved Regions and 9 Hypervariable Regions [10]. The conserved regions have little difference between bacteria [11]. The hypervariable region has the specificity of genus or species, which was with certain differences depending on the kinship [10]. Therefore, 16S rDNA could be used as a characteristic nucleic acid sequence for revealing biological species, which was considered to be the most suitable index for bacterial phylogeny and classification identification [12]. As early as 2005, Lenkinski et al. [13] incubated the stool sample extract, and found that the intestinal flora of premenopausal breast cancer patients was significantly different from that of normal women, the number of *Escherichia coli*, *A. aerobicus*, *Lactobacilli* and non-fermenting bacteria increased significantly. With the development of gene sequencing technology, the research on intestinal flora has entered a new stage. Flores et al. [14] used 16S  RNA sequencing technology to study the difference of intestinal flora between 48 postmenopausal breast cancer patients and 48 normal women. The results showed that compared with normal women, postmenopausal breast cancer patients not only decreased the alpha diversity of the intestinal flora, but also significantly changed their beta diversity. Compared with controls, the number of bacteria in the gastrointestinal tract of the postmenopausal breast cancer patients, *Clostridium* and *Microbacteria* increased significantly, while the number of bacteria in the genus *Derea* was significantly reduced [15]. The results suggested that the intestinal microflora may affect the onset of breast cancer through estrogen-dependent signaling pathways. The study of Santhanam et al. [16] showed that patients in higher the histological grade was always with greater the number of *Bract bacteria* in the gastrointestinal tract.

In addition, tumor biomarkers can reflect the occurrence, development, recurrence and prognosis of tumors, and have important value in early diagnosis, evaluation, prognosis monitoring and new drug development [17]. Metabolomics techniques can detect the metabolic profile of tumors by modern analytical methods, screen metabolic biomarkers and detect abnormal changes in their living organisms [18]. The technology has made good progress in early screening, metastasis and recurrence, efficacy evaluation and prognosis monitoring of breast cancer [19]. It is expected to provide new ideas for the diagnosis and treatment of breast cancer. Buckendahl et al. [20] performed a metabolomic study of breast cancer based on gas chromatography-time-of-flight-mass spectrometry (GC-TOF-MS) technology and found that sputum metabolism and glyceride metabolism were significantly increased in breast cancer tumor tissues. In addition, Sitter et al. [21] processed High-resolution magic angle spinning - nuclear magnetic resonance (HRMAS-NMR) technology to analyze the metabolic profiles of breast cancer tissues and adjacent tissues, and confirmed that the levels of metabolites such as phosphatidylcholine, phosphorylcholine and choline in cancer tissues were significantly higher than those in adjacent tissues. Meta-analysis of urine samples from breast cancer and healthy controls by Zeng et al. found that high vanillin, 4-hydroxyphenylacetic acid, 5-hydroxyindoleacetic acid and urea were used as biomarkers for breast cancer [22]. Metabolomic analysis of urine samples also showed that high vanillin, 4-hydroxyphenylacetic acid, 5-hydroxyindoleacetic acid and urea could be used as biomarkers for breast cancer [23].

Thereby, 16S rDNA and metabolomics study were important for detecting the development and treatment of breast cancer. In order to provide more biomarkers and therapeutic targets for breast cancer, 16S rDNA sequencing and LC-MS metabolomics were firstly combined to assess the alter between gut bacteria and blood metabolites in breast cancer, which might lay a theoretical basis for breast cancer research. Importantly, we firstly reported the inhibitive effects of *Faecalibacterium prausnitzii* for the growth of breast cancer cells through IL-6/STAT3 pathway.

## Results

### 16S rDNA amplicon sequencing analysis

#### OTU analysis and species annotation

The down data obtained by sequencing Illumina HiSeq was spliced and quality controlled to obtain Clean Tags, and then chimera filtering was performed to obtain effective tags which could be used for subsequent analysis. In order to quickly and intuitively reflect the species composition and abundance information in the sample, Heatmap form was used to show the OTC cluster. For the species type results of each sample, the top 10 species of the maximum phase abundance were selected for species classification tree statistics, and the species classification tree statistics were performed. The individual samples were shown in Figure S1, and the grouped species trees are shown in Figure S2. The species classification tree results for either a single sample or grouped species showed that the OTC was mainly clustered in six orders, including 10 genera. Moreover, the sequencing results of top 10 or 30 species were shown in Table S1–2.

#### Relative abundance of species

**Fig. 1:**
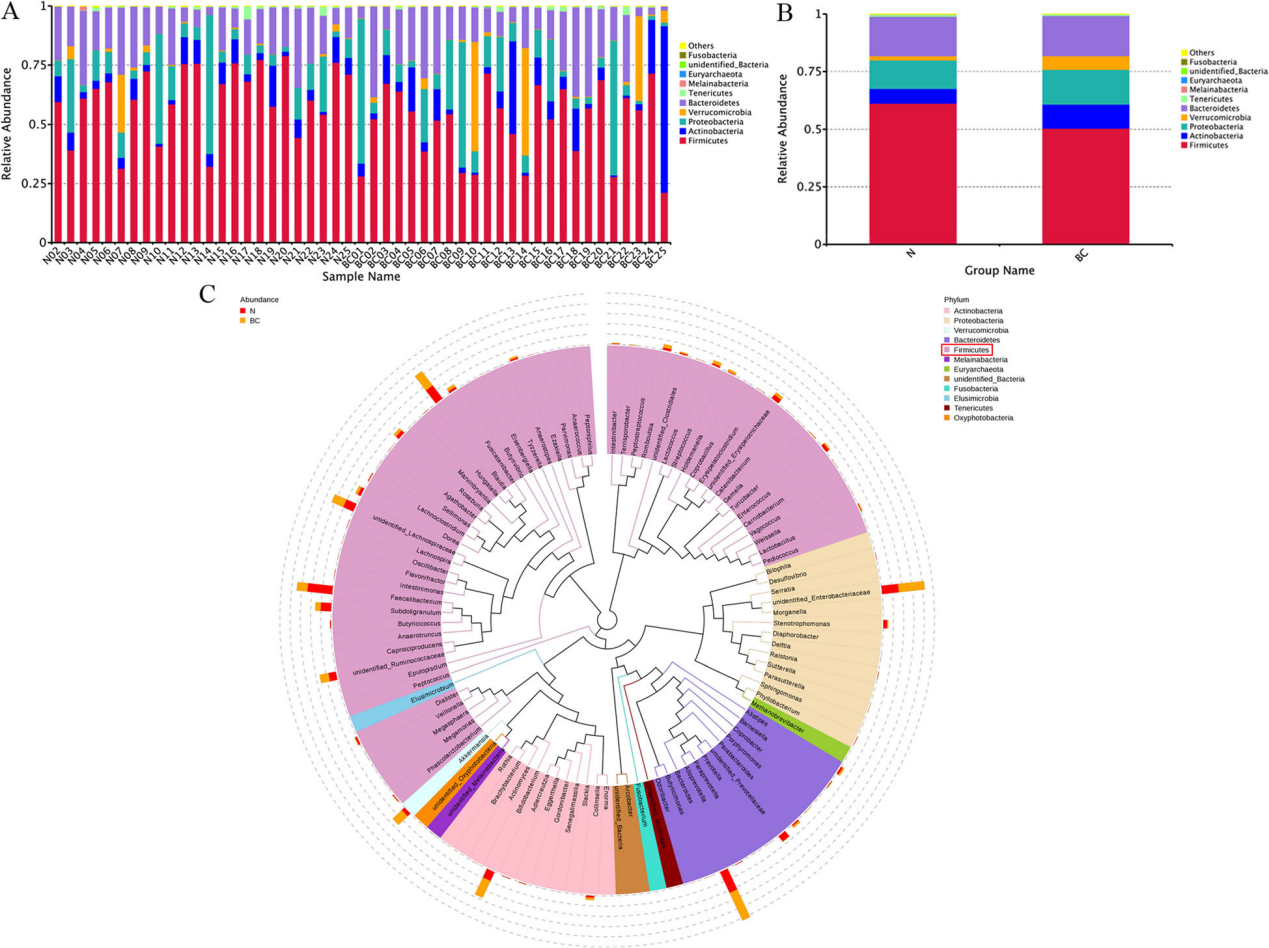
Relative abundance of species. **a** and **b** Relative abundance of species at the phylum level. The abscissa is the sample name or group, and the ordinate indicates the relative abundance. **c** Species evolution tree at Genus. Each color represents a door, and the color of the score indicates its corresponding door

According to the results of the staple food of the species, each species or group of the top ten species with abundance at each classification level (Phylum, Class, Order, Family, Genus) was selected to generate a column-like cumulative graph of relative abundance. As shown in Fig. 1a and b, relative abudance of *Firmicutes* and *Bacteroidetes* were decreased, while relative abundance of *Verrucomicrobla*, *Proteobacteria* and *Actinobacteria* was increased in breast cancer group.

#### Species evolution tree at genus

In order to further study the phylogenetic relationship of genus species, the phylogenetic relationship of representative sequences of TOP100 genera was obtained by multiple sequence alignment, and a phylogenetic tree was constructed. The results are shown in Fig. 1c. The phylum with the greatest number of genuses was *Firmicutes* (as showed with red border in Fig. 1c). In this phylum, there were significant changes in *Subdoligranulum* and *Faecallbacterium*.

#### Alpha diversity

**Fig. 2:**
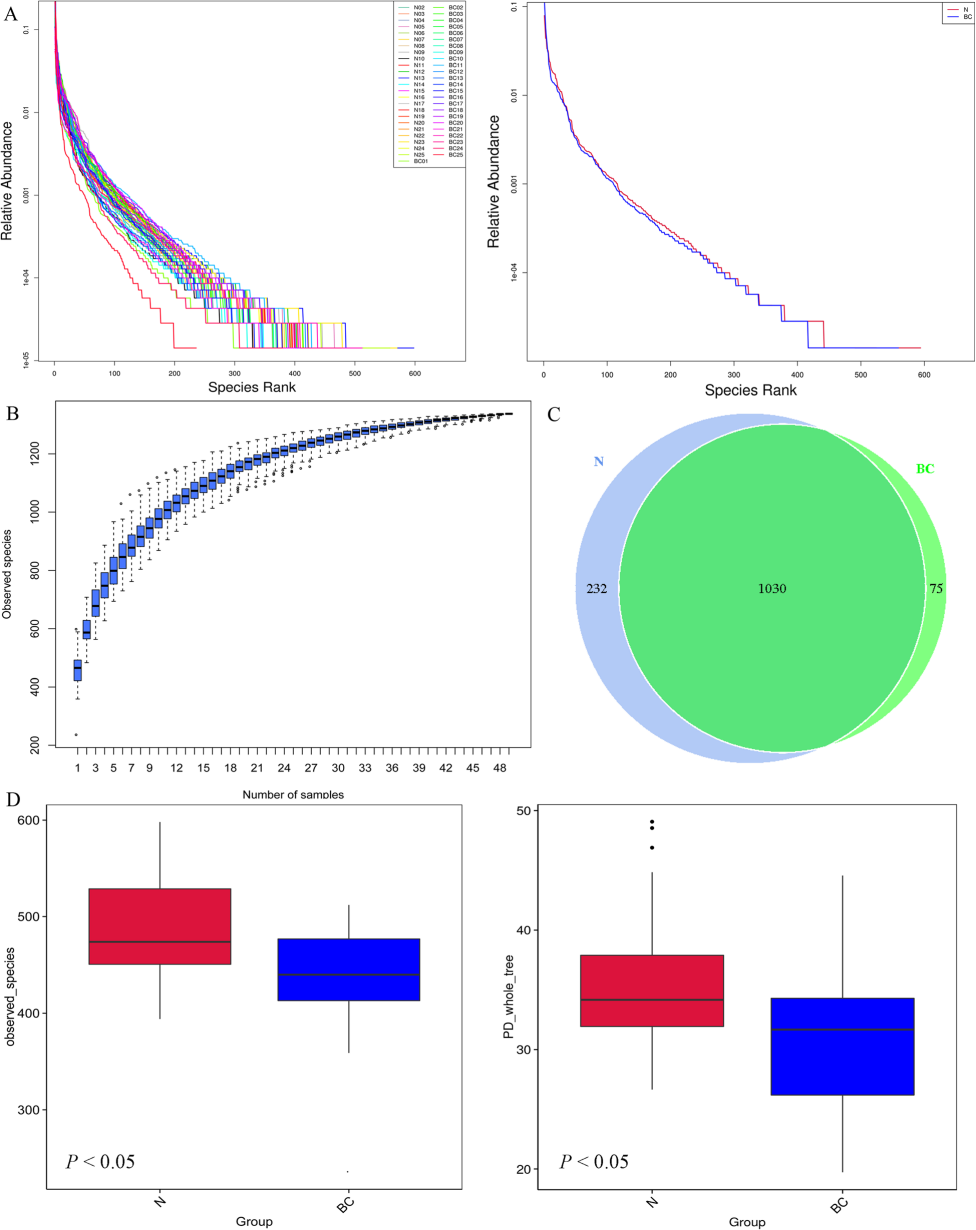
Alpha diversity **a** Hierarchical clustering curves for different samples and different groups. The curves numbered the OTUs in the sample in order of relative abundance from large to small, and then took the ranking number of OTUs as the abscissa and the relative abundance of OTUs as the ordinate, and connected these points with broken lines. The curves are shown by sample and group analysis, respectively. **b** Species accumulation boxplot. The abscissa represents the sample size and the ordinate represents the number of OTU after sampling. The results reflect the rate of new OTU under continuous sampling. **c** Venn graph of OTUs clustering. Venn graph shows the common and unique OTUs between the different groups. A. total common 1030 OTUs were obtained. A total of 75 OTUs was special for breast cancer and 232 OTUs was special for normal group. **d** Observed and PD-whole-tree indexes box between groups. The two box plots show the median species diversity within the group. Alpha-diversity indice is significantly higher in the (normal) N group patients compared with in the (breast cancer) BC group patients (*P* < 0.05)

The Alpha diversity analysis index of different samples at the 97% consistency threshold was counted, and the rationality of the sequencing data of the study was confirmed by the dilution curve. More data only produced a small number of new species. The rank abundance reflected the relative abundance of samples. In the horizontal direction, the richness of the species is reflected by the width of the curve. The higher was the species richness, the larger the span of the curve on the horizontal axis was. In the vertical direction, the smoothness of the curve reflects the degree of uniformity of the species in the sample. The smoother the curve was, the more uniform the species distribution was (Fig. 2a). In addition, in the analysis results of species accumulation boxplot, the species in the environment would not increase significantly with the increase of the sample size, indicating that the sampling was sufficient and data analysis could be performed (Fig. 2b). The Venn diagram was drawn based on the results of the OTUs cluster analysis. As shown in Fig. 2c, total common 1030 OTUs were obtained, such as OUT-31, OUT-560 and OUT-141. A total of 75 OTUs was special for breast cancer, such as OUT-1198, OUT-1111 and OUT-1149. In the analysis of the differences between the Alpha diversity indices, the observed-species and PD-whole-tree were taken as an example, and there were significant differences between the two groups (Fig. 2d). These data suggest that Alpha-diversity of the gut microbiome, defined as the number of species present within each gut sample, was significantly higher in the (normal) N group patients compared with in the (breast cancer) BC group patients (*P* < 0.05 for the two Alpha-diversity indices).

#### Beta diversity and network analysis

**Fig. 3:**
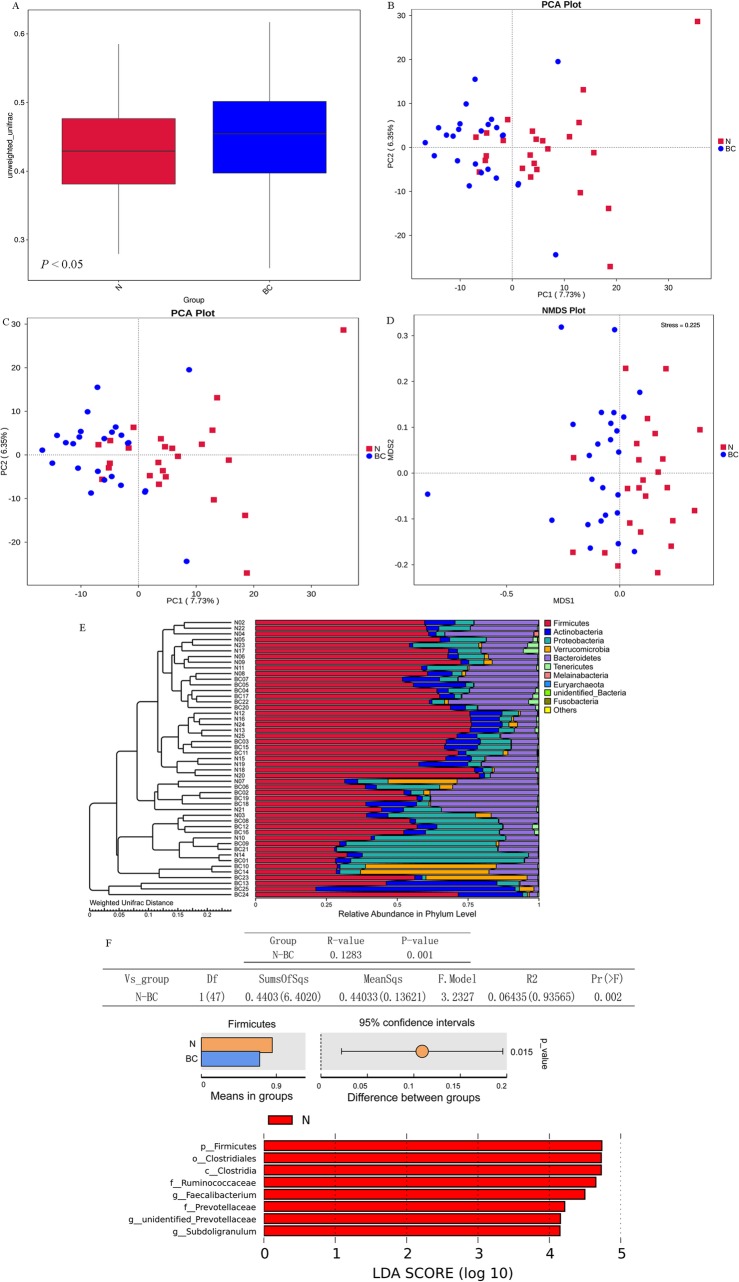
Beta diversity and Network analysis. **a** Box diagram of Beta diversity. The box plot shows the median of sample similarity within the group. Beta diversity indice is significantly lower in the (normal) N group patients compared with in the (breast cancer) BC group patients (*P* < 0.05). **b** PCoA analysis. The abscissa represents a principal component, the ordinate represents another principal component, and the percentage represents the contribution of the principal component to the sample difference. Each point in the figure represents a sample, and samples of the same group are represented by the same color. **c** PCA analysis. The abscissa represents the first principal component and the percentage represents the contribution of the first principal component to the sample difference. The ordinate represents the second principal component and the percentage represents the contribution of the second principal component to the sample difference. Each point in the figure represents a sample, and samples of the same group are represented by the same color. In a PCA graph with clustering circles, clustering circles are added with grouping information. **d** Non-Metric Multi-Dimensional Scaling analysis. Each point represents a sample, and the distance between the points indicates the degree of difference, and the samples of the same group are represented by the same color. When Stress is less than 0.2, NMDS can accurately reflect the degree of difference between samples. D UPGMA clustering tree. **e** Anosim analysis, permutational MANOVA analysis, t-test analysis and LEfSe

Difference analysis between groups of Beta diversity showed significant differences in Beta diversity among groups (Fig. 3a). The results of PCoA and PCA showed that samples in different group were with different community structure and community composition (Fig. 3b and c). Besides, Non-Metric Multi-Dimensional Scaling analysis showed that there were significant differences in community distribution of different samples (Fig. 3d). By unweighted pair-group method with arithmetic mean analysis, relative abundance distribution of species at the Phylum level was mainly *Firmicutes*, *Actinobacteria*, *Proteobaceria* and *Verrucomicrobia* (Fig. 3e). The results of Anosim analysis, permutational MANOVA analysis, t-test analysis of species differences between groups, and LEfSe showed that there were differences in community structure between groups (Fig. 3f). As shown in Figure S3, the network with 75 nodes was constructed. All nodes belonged in 7 phylums, including *Firmicutes*, *Proteobacteria*, *Bacteroidetes*, *Fusobacteria*, *Melainabacteria*, *Actinobacteria* and unidentified bacteria. Thereinto, *Agathobacter*, *Peptostreptococcus*, *Anaerostipes* and *Parvimonas* were hub nodes with higher degree, which were closely related with other bacteria.

### Bioinformatics analysis of differential metabolites

The system stability of the project was analyzed and evaluated by using QC sample spectrum comparison and PCA analysis. The response intensity and retention time of each chromatographic peak almost overlapped, indicating that the variation caused by instrument error was small during the whole experiment. QC samples were tightly clustered in positive and negative ion mode, indicating that the experiment was reproducible. Therefore, the differences in metabolic profiles obtained in the trial could reflect the biological differences between the samples themselves.

#### Typical metabolic profile of each group of samples

**Fig. 4:**
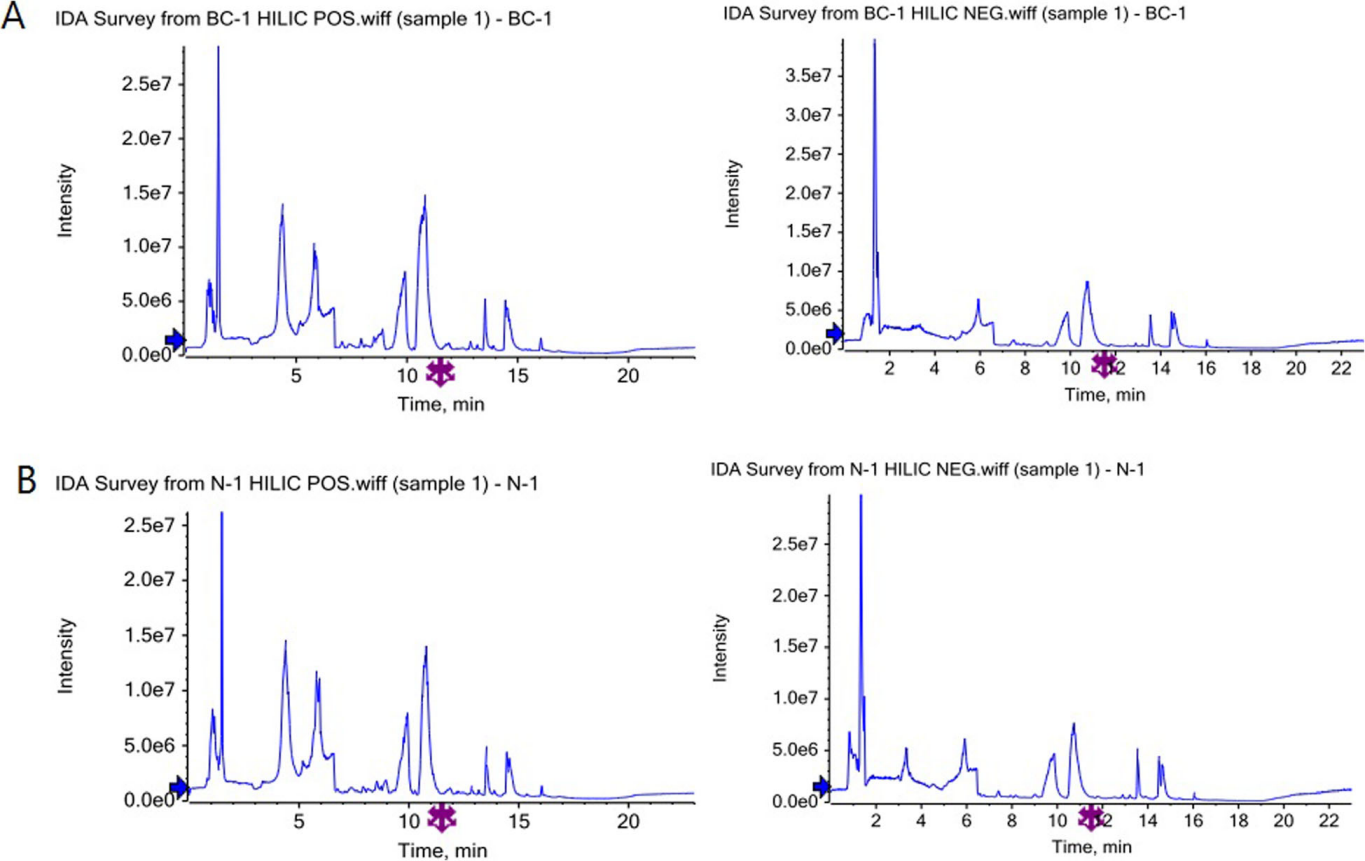
Typical metabolic profile of each group of samples A. Positive and negative ion mode TIC map of BC-1 sample B. Positive and negative ion mode TIC map of N-1 sample

Typical TIC map was obtained after analysis of the sample by UHPLC-Q-TOF MS, and shown in Fig. 4.

#### Analysis of significant difference metabolite

**Fig. 5:**
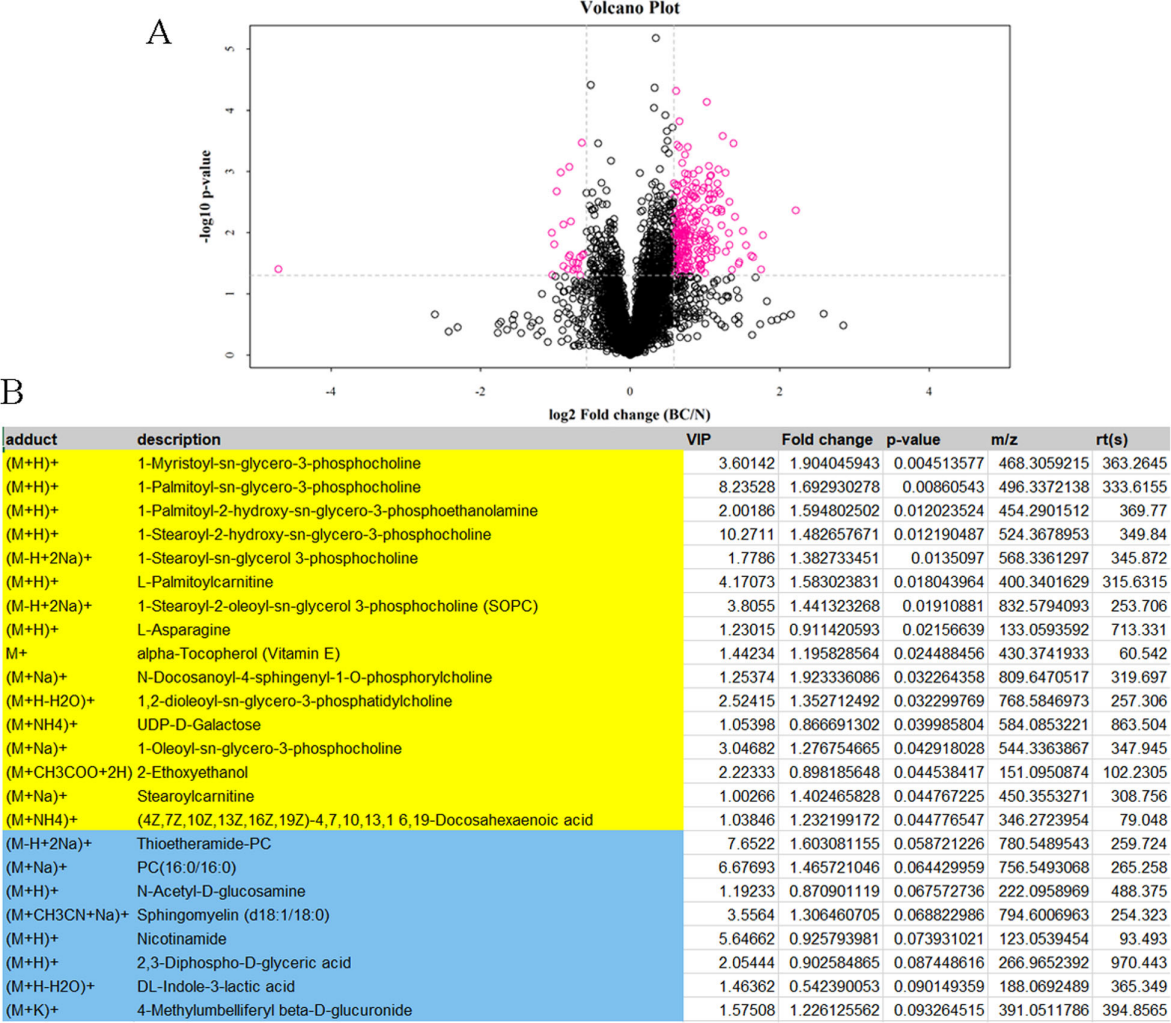
Analysis of significant difference metabolite. **a** Volcano Plot in positive ion mode. The Volcano Plot shows the volcano diagram of the positive ion mode data of the sample group, in which the red dot is fold change > 1.5 and *P* value < 0.05 metabolites, namely the differential metabolites screened by univariate statistical analysis. **b** Difference metabolites identified by positive ion mode. The yellow table shows metabolites with significant difference (multi-dimensional statistical analysis of VIP > 1 and univariate statistical analysis of *P* value < 0.05). The blue table shows differential metabolite (VIP > 1 with 0.05 < *P* value < 0.1)

After pretreatment, PCA was processed to reflect the variability between groups and within groups. The PCA model parameters mainly refer to the value of R2X. The closer R2X is to 1, the model is more stable and reliable. In this study, the R2X value of QC and BC-N group in positive and negative ion mode was 0.295, 0.31, 0.224 and 0.229, respectively. The evaluation parameters of the PLS-DA model in BC-N group were calculated (positive ion mode: R2Y = 0.993, Q2 = 0.542; negative ion mode: R2Y = 0.763, Q2 = 0.125). Besides, OPLS-DA model was also constructed. Model evaluation parameter in BC-N group was also obtained (positive ion mode: R2Y = 0.804, Q2 = − 0.098; negative ion mode: R2Y = 0.763, Q2 = − 0.15). Univariate analysis visually showed the significance of metabolite changes between the two samples (Fig. 5a). According to the Variable Importance for the Projection (VIP) of the OPLS-DA model, the influence of the expression pattern of each metabolite on the classification and discrimination of each group of samples was measured, and the biologically significant differential metabolites were mined. This experiment used VIP > 1 as the screening standard to initially screen out the differences between the groups. As the results, differences identified by positive ion mode were shown in Fig. 5b. Thereinto, 1-myristoyl-sn-glycero-3-phosphocholine, 1-palmitoyl-2-hydroxy-sn-glycero-3-phosphoethanolamine and 1-stearoyl-2-hydroxy-sn-glycero-3-phosphocholine were different with lower *P* value.

#### Bioinformatics analysis of differential metabolites

**Fig. 6:**
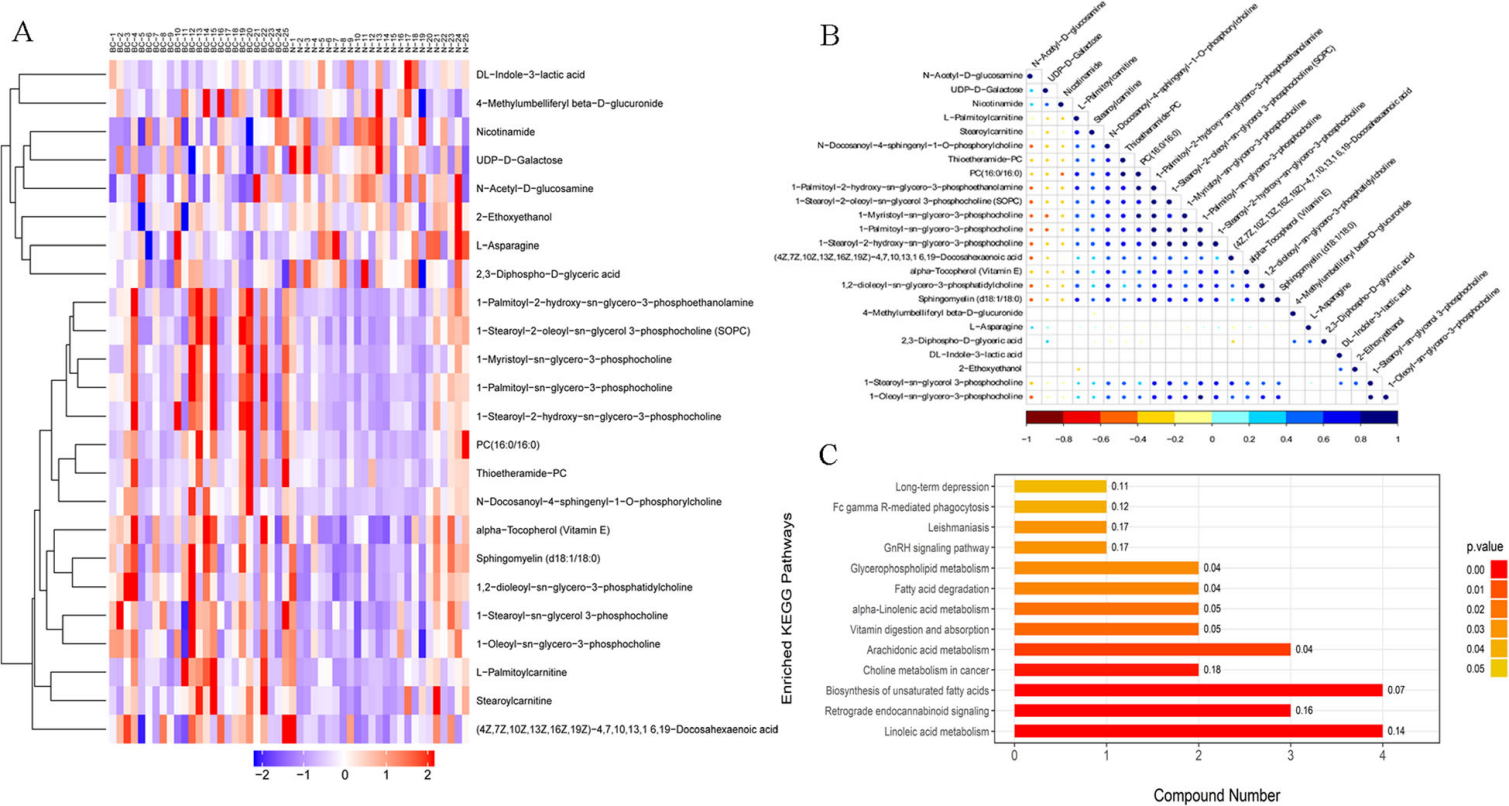
Bioinformatics analysis of differential metabolites. **a** Significant difference metabolite hierarchical clustering results in positive ion mode. **b** Relevance among significant difference metabolites. **c** KEGG pathway enrichment analysis of differentially expressed metabolites

In order to evaluate the rationality of candidate metabolites and to more fully and intuitively show the relationship between samples and the differences in expression patterns of metabolites in different samples, hierarchical clustering was used for significant differential metabolite. Significant difference metabolite hierarchical clustering results in positive ion mode were shown in Fig. 6a. In addition, there was relevance among significant difference metabolites (Fig. 6b). The KEGG pathway enrichment analysis of differentially expressed metabolites by Fisher’s exact test showed that significant changes were observed in important pathways such as linoleic acid metabolism, retrograde endocannabinoid signaling, biosynthesis of unsaturated fatty acids, choline metabolism in cancer and arachidonic acid metabolism (Fig. 6c).

### Correlation analysis

**Fig. 7:**
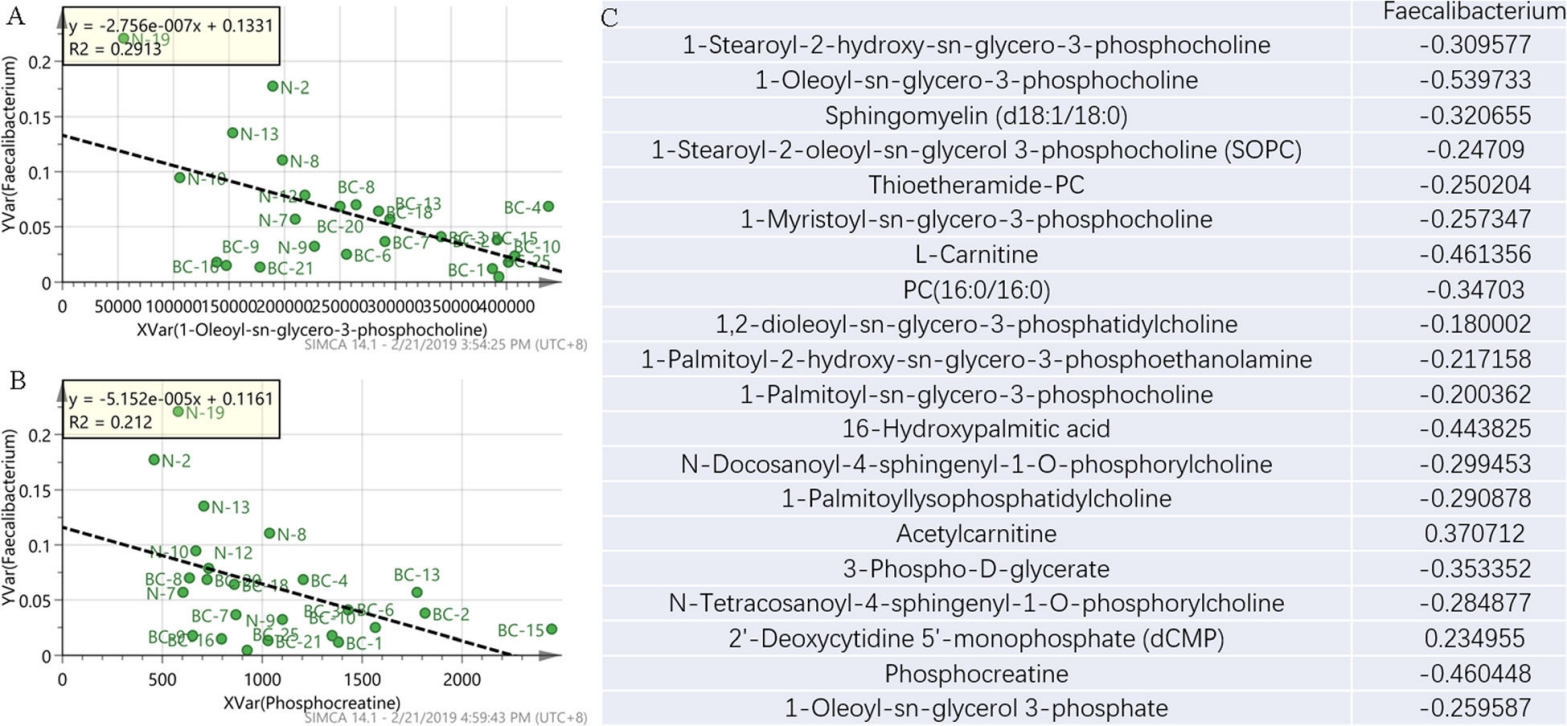
Correlation Analysis of *Faecalibacterium* and phospholipid metabolites. **a** Correlation coefficient between *Faecalibacterium* and 1-Oleoyl-sn-glycero-3-phosphocholine. **b** Correlation coefficient between *Faecalibacterium* and phosphocreatine. **c** Correlation coefficient between *Faecalibacterium* and some phospholipid metabolites

The (breast cancer) BC_vs_(normal) N group had a clear distinction between metabolomics and microbial diversity, and a total of 49 significantly different flora and 26 significantly different metabolites were screened. Through the Spearman statistical analysis method, the correlation between the two was calculated. No significant positive or negative bacteria-metabolites correlations were found. The R language and Cytoscape analysis software provide in-depth data mining between the flora-metabolites, the flora-bacteria, and the metabolite-metabolites from multiple perspectives. Correlation between significant differential flora and significant differential metabolites is presented in the form of a correlation coefficient matrix heat map (Figure S4). The cluster heat map of spearman correlation hierarchical clustering analysis of significant differences in flora and metabolites was shown in Figure S5. Flora in the same cluster of significant differences in metabolites or differences in the genus was with similar correlation patterns. Although there were no positive results in the correlation analysis, we were concerned about the significant reduction of *Faecalibacterium* in breast cancer group. It was negatively correlated with the expression of many phospholipid metabolites, such as 1-stearoyl-2-hydroxy-sn-glycero-3-phosphocholine, 1-Oleoyl-sn-glycero-3-phosphocholine and sphngomyelin (Fig. 7).

### *Faecalibacterium prausnitzii* inhibited IL-6/STAT3 pathway in breast cancer cells

**Fig. 8:**
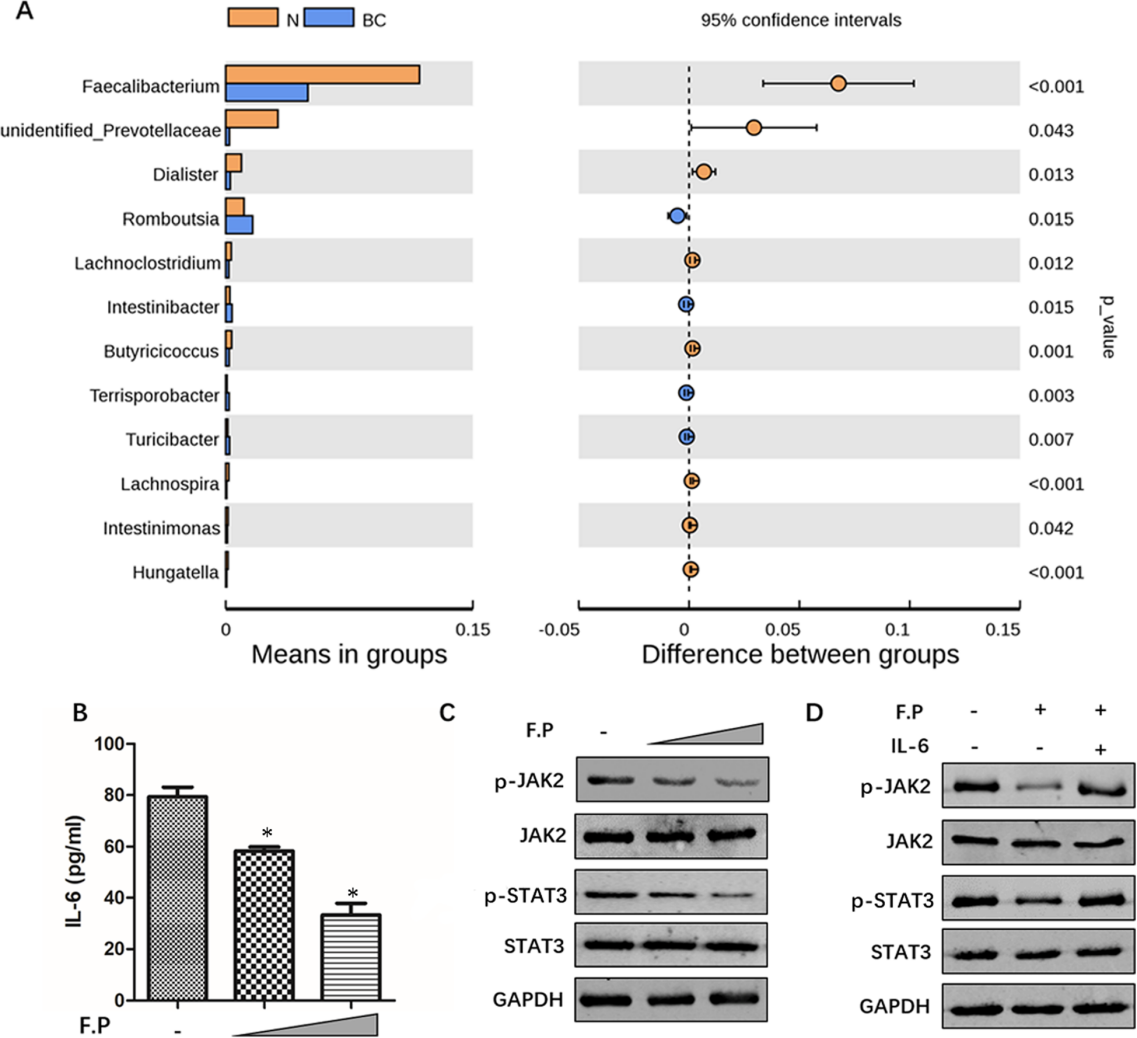
The inhibitive effect of *Faecalibacterium prausnitzii* on IL-6/STAT3 pathway in MCF-7 cells. **a** The abundance gut bacteria difference between breast cancer women group and no-breast cancer women group was detected by 16S rDNA amplicon sequencing analysis. The brown color presents the abundance gut bacteria in no-breast cancer women group and the blue color presents the abundance gut bacteria in breast cancer women group. **b** and **c** MCF-7 cells were incubated with high or low concentration of *Faecalibacterium prausnitzii* (F.P) supernatant, respectively for 72 h. Then cellular medium was collected and detected the secretion level of IL-6. The cells also collected to analyze the protein expression of p-JAK2, p-STAT3, total JAK2 and STAT3. **d** MCF-7 cells were incubated with *Faecalibacterium prausnitzii* (F.P) supernatant or *Faecalibacterium prausnitzii* (F.P) supernatant plus 50 ng/ml recombinant human IL-6, respectively for 72 h. Then cells collected to analyze the protein expression of p-JAK2, p-STAT3, total JAK3 and STAT3. * indicates control vs F.P group, *P* < 0.05

In this study, difference analysis between breast cancer group and normal group of Beta diversity showed that *Faecalibacterium*, a genus of *Clostridiales* and *Firmicutes*, was significant reduced in breast cancer patients compared with normal group (*P* < 0.01, Fig. 8a). This data suggested that *Faecalibacterium* may be helpful for the prevention of breast cancer and the reduction of *Faecalibacterium* promote the development of breast cancer. However, Whether *Faecalibacterium* can inhibit the growth of breast cancer is still unclear. *Faecalibacterium prausnitzii,* the most well-known species of *Faecalibacterium*, is one of the most abundant and most important commensal bacteria in human intestinal microbes. As shown in Fig. 8b-c and Figure S6A, the incubation with *Faecalibacterium prausnitzii* supernatant could inhibit the secretion of IL-6 and the phosphorylation of JAK2/STAT3 in breast cancer cell line MCF-7. In addition, as the concentrations of *Faecalibacterium prausnitzii* supernatant increased, the inhibition is more remarkable. To further confirm whether IL-6 was regulated by *Faecalibacterium prausnitzii* to affect JAK2/STAT3 expression, we used *Faecalibacterium prausnitzii* supernatant and recombinant human IL-6 to incubate MCF-7 cells at the same time. Western blot results indicated that although *Faecalibacterium prausnitzii* was able to inhibit the phosphorylation of JAK2/STAT3, reduced phosphorylation of JAK2/STAT3 caused by *Faecalibacterium prausnitzii* disappeared after the addition of recombinant human IL-6 (Fig. 8d and Figure S6B). These assays indicated that the effect of *Faecalibacterium prausnitzii* on JAK2/STAT3 was mediated at least in part by IL-6 and the anti-tumor effect of *Faecalibacterium prausnitzii* may be through inhibition of the IL6/STAT3 pathway.

### The growth inhibition of breast cancer cells induced by *Faecalibacterium prausnitzii* was mediated by IL-6

**Fig. 9:**
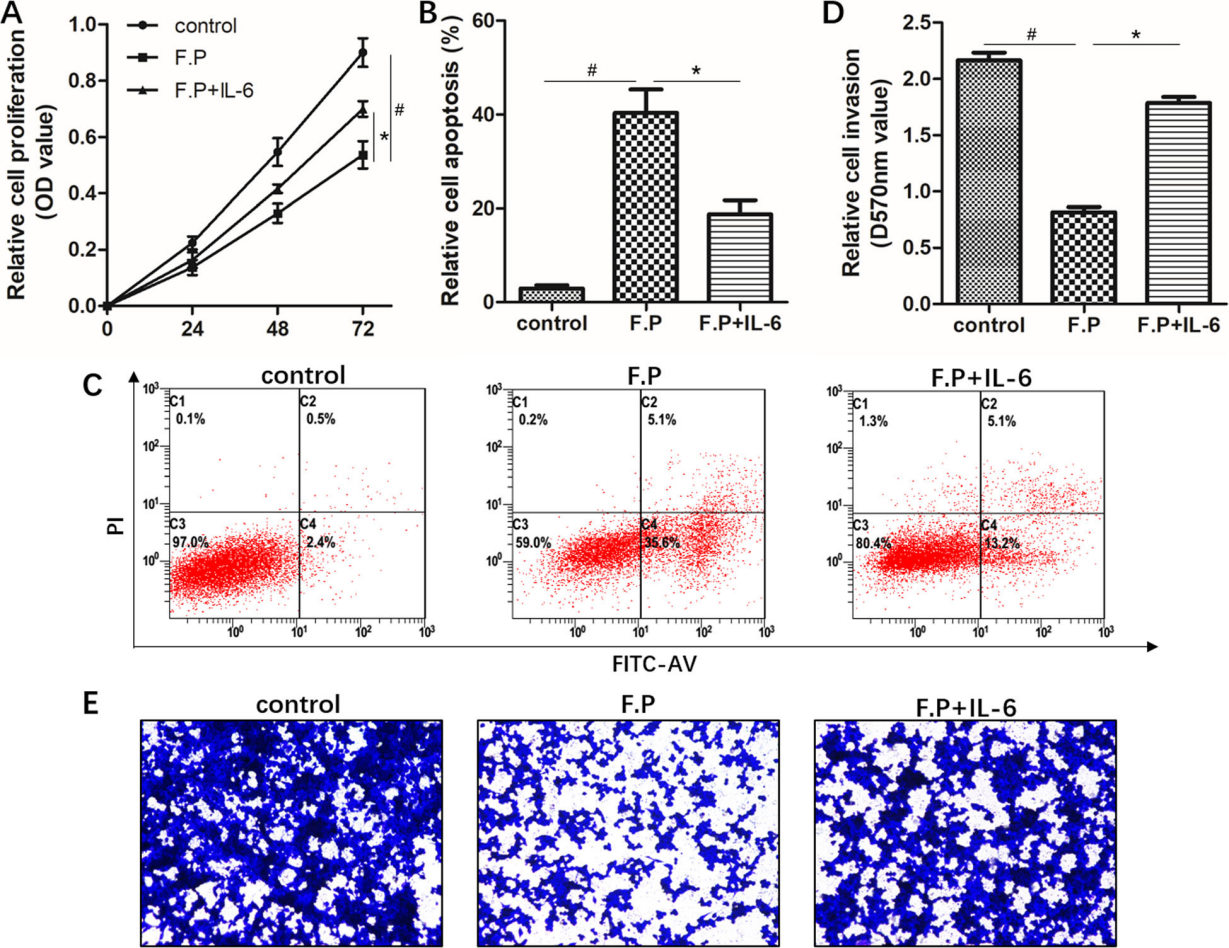
The inhibitive effect of *Faecalibacterium prausnitzii* on the growth of MCF-7 cells through IL-6. MCF-7 cells were subjected to proliferation usingCCK-8, apoptosis and invasion assays as described in the text (see Materials and Methods). MCF-7 cells were incubated with DMEM medium as control, *Faecalibacterium prausnitzii* supernatant (F.P), and *Faecalibacterium prausnitzii* supernatant plus 50 ng/ml recombinant human IL-6 (F.P + IL-6) for 72 h, respectively. Then CCK-8 (**a**), apoptosis (**b** and **c**) and invasion (**d** and **e**) assays were performed and the data was analyzed. # indicates control vs F.P group, *P* < 0.05; *indicates F.P vs F.P + IL-6 group, *P* < 0.05

Here, we studied anti-tumor effect and mechanism of *Faecalibacterium prausnitzii* in breast cancer cells. To confirm that *Faecalibacterium prausnitzii* inhibited the growth of breast cancer cell line MCF-7 through IL-6, recombinant human IL-6 was used to exogenously disrupt the effect of *Faecalibacterium prausnitzii* for IL-6 (Fig. 9). Firstly, it is noteworthy that the proliferation and invasion of MCF-7 cells were suppressed and the apoptosis was promoted significantly by *Faecalibacterium prausnitzii* supernatant. Whereas the above effects were attenuated remarkably after the addition of recombinant human IL-6. These data suggested that *Faecalibacterium prausnitzii* can inhibit the growth of breast cancer cells and these inhibitive effects was mediated in part by IL-6.

## Discussion

In previous study, diet, intestinal microflora and estrogens were confirmed to interact with each other and affect the onset of breast cancer [24]. In animal experiments, it has also been found that breast cancer could be prevented by improving the composition of the fecal microbiota and repairing the intestinal barrier function [25]. Besides, high-throughput sequencing analysis also showed that *Bcatroidetes* Phylum bacteria and *Firmicutes* were significantly different between breast cancer patients and controls [26]. Flora-metabolites and flora-bacteria were with close relationship, but no reports were published.

In order to provide more biomarkers and therapeutic targets for breast cancer, 16S rDNA sequencing and LC-MS metabolomics were firstly combined to assess the interaction between gut bacteria and blood samples of breast cancer. As the results, the BC_vs_N group had a clear distinction between metabolomics and microbial diversity, and a total of 49 significantly different flora (such as *Geobacter*, *Thermoanaerobaculum*, *Shewanella*, *Gluconobacter* and *Domibacillus*) and 26 significantly different metabolites (such as choline, 1-Palmitoyl-sn-glycero-3-phosphocholine, L-Carnitine and indolelactic acid) were screened. Through the Spearman statistical analysis method, the correlation between the two indexes was calculated. Lipid upregulation is found in plasma of breast cancer patients, especially phosphorocholine. Interestingly, *Faecalibacterium* is the most important differential genus and reduced significantly in breast cancer patients, which is negatively correlated with various phosphorylcholines. The R language and Cytoscape analysis software provide in-depth data mining between the flora-metabolites, the flora-bacteria, and the metabolite-metabolites from multiple perspectives.

Clinical studies have shown that the intestinal flora could affect reabsorption of free hormones and metabolism of estrogens and further link to breast cancer [24]. *Magnesium glucuron* in the intestinal flora promoted re-absorption of estrogen, and estrogen levels were closely related to the development of breast cancer [27]. Thereby, the intestinal flora might affect occurrence and development of breast cancer by interacting with estrogen in the human body. Wang et al. [28] selected stool samples from patients with early breast cancer and used 16sRNA gene detection technology to detect the composition of the flora. The results showed that the higher the histological grade of the tumor, the more the number of Bract bacteria in the gastrointestinal tract. In addition, the study also observed significant differences in the number of patients with early breast cancer of different body mass index in the gastrointestinal tract, such as the number of *P. sphaericus*, thick-walled bacteria, and *Bacterella*. Similar results were also obtained in this study. *Agathobacter*, *Peptostreptococcus*, *Anaerostipes*, *Parvimonas* and some other genus were significant different between breast cancer patients and controls. The results were similarly with previous studies. Compared with no-breast cancer women, women with breast cancer have more *Enterobacter* and *Staphylococcus* [29]. Interestingly, a well-known member of the *Enterobacteriaceae* family - *E. coli* may be associated with breast cancer [30]. Moreover, there was high abundance of *Bacillus*, *Enterobacter*, and *Staphylococcus* in breast tissue of breast cancer patients. It worth noting that *Peptostreptococcus* is a normal flora of the human mouth, upper respiratory tract, intestines and female reproductive tract. The bacteria of this genus included *P.anaerobius*, *P.magnus*, *P.micros*, *P.asaccharolyticus* and *P.prevotii*. *Streptococcus pneumoniae* increased the biosynthesis of reactive oxygen species (ROS) and cholesterol in cells via regulation of Toll-like receptors Toll 2 (TLR2) and Toll-like receptors Toll 4 (TLR4, [31]). An increase in intracellular cholesterol levels would increase the level of cell proliferation [32]. Chinese study have found that the detection rate of anaerobic bacteria in breast cancer tissues is 65.7%, that in benign tumor tissues is 23.1%, and that in distant breast tissues is 10.4% [33]. Therefore, the high detection rate of anaerobic bacteria suggested that it had some association with breast cancer. The affection of *Parvimonas* was only reported in colorectal cancer, no study researched the relationship between *Parvimonas* and breast. In this study, *Parvimonas* was confirmed significant different between breast cancer patients and control. Thereby, these bacteria might be a target for breast cancer treatment.

Moreover, the metabolomic changes were performed on the samples by metabolomics method based on HILIC UHPLC-Q-TOF MS in this experiment. The quality control experiment showed that the instrument analysis system of this experiment had good stability, and the test data was stable and reliable. The differences in metabolic profiles obtained in the experiments could reflect the biological differences between the samples themselves. So far, the application of metabolomics in malignant tumors mainly includes early diagnosis of tumors, identification of tumor-associated biomarkers and study of tumor metabolic processes [34]. Previous study confirmed that a large number of small and medium molecular substances increased during the development of breast cancer [35]. The detection and analysis of these small and medium molecular substances will help patients to diagnose the disease and evaluate the effect after chemotherapy [36]. Interestingly, choline, 1-Palmitoyl-sn-glycero-3-phosphocholine, L-Carnitine and indolelactic acid were confirmed differently metabolism in breast cancer patients in this study. Thereinto, choline was an important nutrient in food [37]. A study supported by the National Institutes of Health (NIH) showed that choline could reduce the risk of breast cancer by 24% [38]. In 2010, Eliyahu et al. [39] specifically referred that phosphocholine could be regarded as an important biomarker for breast cancer, it was important for regulating the expression of choline kinase genes and some specific transporters. Besides, under the ultra-rapid cooling program, the cryopreservation efficiency of human breast cancer cells frozen by L-carnitine was significantly better than the existing cryoprotectant, such as Dimethylsulfoxide (DMSO) [40]. Furthermore, orally administrable dietary supplement with L-carnitine could control cancer related fatigue in breast cancer patients [41]. However, Indolelactic acid in breast cancer has not been researched so far. It might be a new potential metabolomic biomarker for diagnose of breast cancer. Thereby, most of above metabolomics could reflect the development of breast cancer.

Interestingly, *Faecalibacterium* is the most important differential genus, which is negatively correlated with various phosphorylcholines in this study. In previous studies, it has been confirmed to play important role in various cancers. For example, Behnoush et al. [42] verified that *Faecalibacterium prausnitzii* improved secreting extracellular vesicles process, up-regulated the expression of anti-inflammatory cytokines (IL-10, TGF-β2 and IL-1Ra), and down-regulated the expression of pro-inflammatory cytokines (IL-6, TNF-α and TNF-β) in lung cancer disease. This data was similar with ours that *Faecalibacterium prausnitzii* could inhibit the expression of IL-6 in breast cancer cells. Moreover, losing of *Faecalibacterium prausnitzii* could induce dysbiosis of anti-inflammatory mechanism, which was a critical feature of Crohn’s disease [43]. Patients with Crohn’s disease have an increased risk of colorectal cancer with prolonged disease duration [44]. Interestingly, *Faecalibacterium prausnitzii* and its supernatant could also regulate the expression of IL-10 and TNF-α, and then inhibit load of colorectal cancer [45]. Though in previous study the mechanism of *Faecalibacterium prausnitzii* in breast cancer has not been researched, in this study we firstly reported the growth inhibition of breast cancer cells induced by *Faecalibacterium prausnitzii*. Our data showed that compared with in non-breast cancer women group, the abundance of *Faecalibacterium* was decreased significantly in breast cancer women group. It indicated that *Faecalibacterium* may be helpful for the prevention of breast cancer and the reduction of *Faecalibacterium* promote the development of breast cancer. We also found that *Faecalibacterium prausnitzii* supernatant can inhibit the proliferation and invasion and promote the apoptosis of breast cancer cells.

IL-6 is a multifunctional cytokine that was originally determined to be a regulator of immune and inflammatory responses and associated with certain epithelial tumors [46]. Especially, it was reported that serum IL-6 levels correlate with poor survival in patients with hormone receptor positive metastatic breast cancer [47]. IL-6 binds to a heterodimeric receptor containing the ligand-binding IL-6 chain and the common cytoline receptor signal-transducing subunit gp130[48]. IL-6 receptor engagement leads to activation of JAK2 of tyrosine kinases, which then stimulate pathways involving phosphatidylinositol STAT3 [49]. In this study, we found that *Faecalibacterium prausnitzii* could inhibit the secretion of IL-6 and the phosphorylation of JAK2/STAT3 in breast cancer cells. Maybe, it was one reason for the growth inhibition of breast cancer cells induced by *Faecalibacterium prausnitzii.*

## Conclusion

In our study, combined 16S rDNA sequencing and LC-MS metabolomics was a useful method to assess the interaction between gut bacteria and blood samples of breast cancer. Flora-metabolites combined with the flora-bacteria (such as *Faecalibacterium* combined with phosphorocholine) might a new detection method for breast cancer. Importantly, the abundance of *Faecalibacterium* was decreased in breast cancer women and we also found that *Faecalibacterium prausnitzii* supernatant can suppress the growth of breast cancer cells through the inhibition of IL-6/STAT3 pathway. It indicated that *Faecalibacterium* may be helpful for the prevention of breast cancer and the reduction of *Faecalibacterium* promote the development of breast cancer.

## Methods

### Fecal and blood samples

Total 25 breast cancer patients and 25 patients with benign breast disease as controls from Apr 2017 to Sep 2018 in the 940th Hospital of Joint Logistics Support Force of Chinese People’s Liberation Army were included in this study. All patients or their families signed informed consent, and the experiments were approved by ethics committee of our hospital. All patients were newly diagnosed without any medication or radiation therapy. None of the study subjects had diarrhea, diabetes, ulcerative colitis, Crohn’s disease, or other infectious diseases. No subjects took antibiotics, steroid hormones, Chinese herbal medicine, or probiotics such as yogurt during the 3 months before fecal sample collection. The fecal and blood samples were paired obtained from above patients. Fecal samples were freshly collected from individuals and transported to the laboratory on ice. Samples were stored at − 80 °C until detection. 16S rDNA sequencing, LC-MS metabolomics were performed as instructions by Shanghai Applied Protein Technology. The Fecal sample of one control was unavailable through 16S rDNA sequencing.

### 16S rDNA amplicon sequencing analysis

#### DNA extraction and polymerase Chain reaction (PCR) amplification

The genomic DNA of the sample was extracted by cetyltriethylammnonium bromide (CTAB) method, and then the purity and concentration of the DNA were detected by agarose gel electrophoresis. Diluted genomic DNA was used as a template, PCR was performed, and the PCR product was detected by 2% agarose gel electrophoresis, and the product was recovered using the gel recovery kit provided by Qiagen for the target strip. The library was constructed using the TruSeq® DNA PCR-Free Sample Preparation Kit. The constructed library was quantified by Qubit and Q-PCR. After the library was qualified, it was sequenced by Illumina HiSeq.

#### Data analysis process and method

Raw data was obtained, first spliced and filtered to obtain clean data. OTUs (Operational Taxonomic Units) clustering and species classification analysis were then performed based on valid data. Species annotations were made for the representative sequences of each OTU, and the corresponding species information and species-based abundance distribution were obtained. At the same time, OTUs were analyzed for abundance, Alpha diversity calculations, Venn plots, and petal plots to obtain species richness and uniformity information within the sample. Common and unique OTUs information between different samples or groups were also obtained. On the other hand, multiple sequence alignments could be performed on OTUs and phylogenetic trees could be constructed, and community structure differences between different samples and groups could be further obtained. The results were demonstrated by Principal Coordinate Analysis (PCoA) and Principal Component Analysis (PCA), Non-metric multidimensional scale (NMDS) and other dimensionality reduction maps and sample clustering trees. In order to further explore the differences in community structure between grouped samples, statistical analysis methods such as T-test, LEfSe, Anosim and MRPP were used to test the species composition and community structure of the grouped samples. The detail data analysis method was as follows.

##### Sequencing data processing

Each sample data was separated from the off-machine data according to the Barcode sequence and the PCR amplification primer sequence. After trimming the Barcode and primer sequences, FLASH (V1.2.7, http://ccb.jhu.edu/software/FLASH/) was used to splice the reads of each sample, and the splicing sequence was obtained as Raw Tags. The Raw Tags were stitched together under require strict filtering to obtain clean tags. Intercept and filter was processed according to the Tag quality control process of Qiime (V1.9.1, http://qiime.org/scripts/split_libraries_fastq.html). The Tag obtained after the above processing needs to be processed to remove the chimera sequence, and the Tags sequence was compared with the database (Gold database, http://drive5.com/uchime/uchime_download.html) to detect the chimeric sequence, and finally remove the chimeric sequences to obtain the final Effective Tags.

##### OTU clustering and species annotation

Ufse software (Uparse v7.0.1001, http://drive5.com/uparse/) was used to cluster all the Effective Tags of all samples. By default, the sequences were clustered into OTUs with an agreement of 97%. At the same time, the representative sequence of OTUs was selected. According to its algorithm principle, the sequence with the highest frequency in OTUs was selected as the representative sequence of OTUs. Species annotation was performed on the OTUs representative sequence, and species annotation analysis was performed using the Mothur method and the SSUrRNA database of SILVA (http://www.arb-silva.de/) (threshold was 0.8–1). Taxonomic information was obtained and the community composition of each sample at each classification level was statistically calculated. Fast multi-sequence alignment was performed using MUSCLE (Version 3.8.31, http://www.drive5.com/muscle/) software to obtain a systematic relationship of all OTUs representative sequences. Finally, the data of each sample were homogenized, and the sample was homogenized with the least amount of data in the sample. Subsequent Alpha diversity analysis and Beta diversity analysis were processed based on the data after homogenization.

##### Alpha diversity

Observed-species, Chao1, Shannon, Simpson, ACE, Goods-coverage, PD_whole_tree index was calculated by Qiime software (Version 1.9.1), while the dilution curve, the Rank abundance curve, and the species accumulation curve was drawn by the R software (Version 2.15.3). Difference analysis between groups of Alpha diversity index was processed using R software.

##### Beta diversity

The Unifrac distance was calculated using Qiime software (Version 1.9.1), and the UPGMA sample clustering tree was constructed. PCA, PCoA and NMDS maps were drawn using R software (Version 2.15.3). The R software’s ade4 package and ggplot2 software package were used for PCA analysis. The PCoA analysis was processed using the W software’s WGCNA, stats and ggplot2 software packages, and the NMDS analysis was undertaken by the vegan software package of R software. If there were only two groups, the T-test and Wilcox tests were used while the Tukey test and the wilcox test of the agricolae package are used for comparison of more than two groups. The significance test of community structure differences between groups was analyzed by Anosim and Adonis, using the anosim function of the R vegan package and the adonis function, respectivley. Differences between groups were analyzed using T-test and LEfSe analysis. T-test was processed by R software. LEfSe analysis was processed with the default setting of LDA Score = 4.

##### Network construction analysis

The co-occurrence network diagram provides a new perspective for studying the community structure and function of the complex micro-environment environment. After calculating the Spearman correlation coefficient for all samples and obtaining the species correlation coefficient matrix, the filtering conditions are set as follows: (1) removing the connection whose correlation coefficient is less than 0.6; (2) filtering out the node self-join; (3) removing the node seal Block the connection less than 0.005% to get the network map.

### LC-MS metabolomics analysis

The HILIC UHPLC-Q-TOF MS technique was combined with the data-dependent acquisition method to perform full-spectrum analysis of the sample, and the first-order mass spectrometry and the second-order mass spectrometry data were obtained simultaneously. Then the peak extraction and metabolite identification of the data were performed by XCMS.

#### Sample pretreatment

After the sample was slowly thawed at 4 °C, 100 μL of the sample was taken, and 400 μL of pre-cooled methanol/acetonitrile solution (2:2, v/v) was added and vortexed. After low temperature sonication for 30 min, − 20 °C for 10 min, centrifuged at 14000 g for 4 min at 4 °C, the supernatant was vacuum dried, and 100 μL of acetonitrile aqueous solution (acetonitrile: water = 1:1, v/v) was reconstituted by mass spectrometry. The sample was reconstituted, vortexed, centrifuged at 14000 g for 5 min at 4 °C, and the supernatant was taken for injection analysis.

#### Chromatography-mass spectrometry

##### Chromatographic conditions

Samples were separated on an Agilent 1290 Infinity LC Ultra High Performance Liquid Chromatography System (UHPLC) HILIC column. Samples were placed in a 4 °C autosampler throughout the analysis. In order to avoid the effects of instrument detection signal fluctuations, continuous analysis of samples is performed in a random order. QC samples were inserted into the sample queue to monitor and evaluate the stability of the system and the reliability of the experimental data.

##### Q-TOF mass spectrometry conditions

Electrospray ionization (ESI) positive and negative ion modes were used for detection. The sample was separated by UHPLC and subjected to mass spectrometry using a Triple TOF 5600 mass spectrometer (AB SCIEX).

##### Data processing

The raw data was converted to a new format (.mzXML) by ProteoWizard, and then the XCMS program was used for peak alignment, retention time correction, and peak area extraction. Metabolite structure identification was processed by a method of accurate mass matching (< 25 ppm) and secondary spectral matching to retrieve a self-built database from the laboratory. For the data extracted by XCMS, the application software SIMCA-P 14.1 (Umetrics, Umea, Sweden) was used for pattern recognition. After Pareto-scaling preprocessing, the data is analyzed by multi-dimensional statistical analysis, including unsupervised PCA, supervised partial least squares discriminant analysis (PLS-DA) and orthogonal partial least squares discriminant analysis (OPLS-DA). Single-dimensional statistical analysis included Student’s t-test and variation multiple analysis; volcano maps was drawn by R software.

#### Univariate statistical analysis

The commonly used univariate analysis methods for differential metabolite analysis between the two groups of samples include coefficient of variation analysis, T-test, and volcano maps that combine the first two methods. Univariate analysis could be used to visualize the significance of metabolite changes between two samples, helping to screen for potential marker metabolites.

#### Significant difference metabolite screening

According to the variable weight value obtained by the OPLS-DA model, the influence intensity and explanatory ability of each metabolite expression pattern on the classification and discrimination of each group of samples were measured, and the biologically significant differential metabolites were mined. In this experiment, the VIP > 1 was regarded as the screening standard, and the differences between the groups were initially screened. Univariate statistical analysis was further used to verify whether the differential metabolites were significant. Metabolites with both multidimensional statistical analysis VIP > 1 and univariate statistical analysis *P* value < 0.05 were selected as metabolites with significant differences. While VIP is > 1 and univariate statistical analysis 0.05 < *P* value < 0.1 was regarded as differential metabolite.

#### Bioinformatics analysis of differential metabolites

##### Cluster analysis of differential metabolite

In order to evaluate the rationality of candidate metabolites and show the relationship between samples and the difference in expression patterns of metabolites in different samples more comprehensively and intuitively, we used qualitative and significant differential metabolite expression to perform Hierarchical Clustering on each group of samples, which helps us to accurately screen the marker metabolites and study the changes of related metabolic processes.

##### Correlation analysis of differential metabolite

Correlation analysis was used in this study to measure the close correlation between significant metabolic differences and further understand the interrelationships between metabolites during changes in biological status.

##### Kyoto encyclopedia of genes and genomes (KEGG) pathway analysis and enrichment of differential metabolites

KEGG pathway analysis and enrichment of differential metabolites were processed. Fisher’s exact test was used to analyze and calculate the significance level of the metabolite enrichment of each pathway to determine the metabolic and signal transduction pathways that receive significant effects.

### Correlation analysis

The relative abundance of 49 populations with significant differences in genus levels (t-test *P* value < 0.05) and expression level of 26 significant differential metabolites of metabolomics analysis (VIP > 1 and t-test *P* value < 0.1) were compiled in a table as an input file for subsequent analysis. The content of association analysis included correlation coefficient calculation, matrix heat map analysis, cluster analysis, correlation network analysis and scatter plot analysis. Based on the spearman correlation analysis method, the correlation between significant differential flora and significant differential metabolites was presented in the form of a correlation coefficient matrix heat map. In order to visually reflect the similarities and differences of the expression patterns of the significantly different flora and the significantly different metabolites, Spearman correlation hierarchical clustering analysis was performed on the significantly different flora and metabolites. The software used was the R 3.4.2 Heatmap package.

### Cell and bacterial culture

The human breast cancer cell line MCF-7 was from ATCC and cultured in DMEM medium supplemented with 10% fetal bovine serum in 5% CO_2_ at 37 °C. The culture medium of MCF-7 cells contained 0.01 mg/ml insulin. *Faecalibacterium prausnitzii* was also form ATCC and grown at 37 °C in an anaerobic incubator containing 97% CO2 and 3% H2. *Faecalibacterium prausnitzii* was cultured in LYHBHI medium containing with Brain-heart infusion (37 g/L), yeast extract (5 g/L), hemin (5 mg/L), supplied with cellobiose (1 g/L), maltose (1 g/L), and cysteine (0.5 g/L). For cellular experiments, bacteria were grown until a stationary phase and counted by bacterial plate count, and centrifuged by 5000 r/min for 5 min. Then, bacterial supernatant was collected and lyophilized. The 3 g lyophilized supernatant can be obtained from 100 mL supernatant. The lyophilized supernatant was diluted with DMEM medium as low concentration (1/5) or high concentration (1/2) for the cellular experiments.

### Western blot and ELISA assays

The cells were collected and lysed in RIPA buffer with protease inhibitors and phosphatase inhibitors (Calbiochem, Darmstadt, Germany). The protein concentration was determined by the Bradford protein assay kit (BioRad). For western blot, 80–100 μg proteins were resolved by SDS-PAGE and transferred to polyvinyl difluoride (PVDF) membranes (Millipore). The blots were probed with the different primary antibodies and species-matched secondary antibodies. Rabbit monoclonal anti-JAK2 (1:1000 dilution; Abcam), rabbit monoclonal anti-phospho-JAK2 (1:1000 dilution; Abcam), mouse monoclonal anti-STAT3 (1:1000 dilution; Abcam), rabbit monoclonal anti-STAT3 (phosphor Y705) (1:1000 dilution; Cell Signaling Technology Inc., USA), rabbit polyclonal anti-GAPDH (1:3000 dilution; BIOS Biotechnology, Beijing, China) were used. The bands were detected using enhanced chemiluminescence (Pierce) or the Odyssey Imaging System (LiCor Biosciences). The cells were incubated with DMEM medium (as control) or different concentration of *Faecalibacterium prausnitzii* (F.P) supernatant, respectively for 72 h. Then cellular medium was collected and detected the secretion level of IL-6. IL-6 was measured using a Human IL-6 ELISA Kit (Abcam, UK) according to the manufacturer’s protocols.

### Cell proliferation, apoptosis and invasion assays

The assays were divided into three groups: control group, *Faecalibacterium prausnitzii* (F.P) supernatant group, F.P supernatant *+* recombinant human IL-6 (Sigma-Aldrich, USA). For the cell proliferation assay, the cells were seeded in 96-well plates (5 × 10^3^ cells per well) for 24 h and were allowed to adhere overnight in regular growth media. Then the culture medium of cells was discarded carefully and 200 μl DMEM medium (control group), *Faecalibacterium prausnitzii* supernatant (F.P group) or F.P supernatant *+* recombinant human IL-6 (F.P + IL-6 group) were added in the each well. After treatment with different groups for 72 h, the relative cell proliferation was measured using the Cell Counting Kit-8 (Dojingdo, Kumamoto, Japan). For the apoptosis assay, we used the Annexin V-FITC Apoptosis detection kit (Dojindo Molecular Technologies, Japan). Cells were collected and washed twice with PBS and then resuspended in 500 μl of staining solution containing fluorescein isothiocyanate (FITC)-conjugated annexin V antibody (5 μl). Subsequent to the staining, the cells were incubated with 10 μl of propidium iodide (PI) for 5 min on ice in the dark. The analyses were performed using a Navios flow cytometer (Beckman Coulter). For the cell invasion assay, the cells were seeded in transwell chambers and the lower culture medium was added with DMEM, F.P supernatant, and F.P supernatant+IL-6. After 3 days, the chamber was removed, PBS was rinsed, and the invasive cells were stained with crystal violet solution, and then decolorized with 33% acetic acid to completely elute the crystal violet. The OD value was measured at 570 nm, which indirectly reflected the cell invasion.

### Statistical analysis for cell experiments

Statistical analyses were performed with GraphPad Prism version 5.0 (La Jolla, CA, USA). Data were analyzed statistically using a one-way analysis of variance (ANOVA) and the independent-samples t test. All data are expressed as the mean ± standard deviation. *P* values of less than 0.05 were considered statistically significant.

## Supplementary information

**Additional file 1: Figure S1** Species classification tree in a single sample**.** Circles of different colors indicate the level of classification. The size of the phase is abundance. The two numbers below the category name indicate relative percentages, the former accounting for the percentage of all species in the sample, and the latter representing the percentage of the sample selected. The classification of the red font indicates that the classification annotation does not exist in the sample, but is present in other analysis samples.

**Additional file 2: Figure S2** Species classification tree in grouped species. Fans of different colors in a circle represent different groups. The size of the sector indicates the proportion of the group’s relative abundance in the classification. The number below the category name indicates the average relative abundance percentage of all groups in the category. The former indicates the percentage of all species and the latter indicates the percentage of selected species.

**Additional file 3: Figure S3** Network analysis. Different nodes represent genus, the node size represents the average relative abundance of the genus, and the nodes of the same gate have the same color. The line thickness between the nodes is positively correlated with the absolute value of the correlation coefficient of the species interaction, and the color and correlation of the line are positively and negatively correlated (red represent positive correlation, blue represent negative correlation).

**Additional file 4: Figure S4** Correlation coefficient matrix heat map. A correlation coefficient matrix heat map of significant differential flora and metabolites

**Additional file 5: Figure S5** Cluster heat map of spearman correlation. The cluster heat map of spearman correlation hierarchical clustering analysis of significant differences in flora and metabolites

**Additional file 6: Figure S6** Quantitative analysis of protein expression. The protein band intensities were quantified with the Odyssey infrared imaging system. Data are presented as the mean ± standard deviation from three independent experiments. A. # indicates control vs high F.P group, *P* < 0.05; *indicates control vs low F.P group, *P* < 0.05. B. # indicates control vs F.P group, *P* < 0.05; *indicates control vs F.P + IL-6 group, *P* < 0.05.

## Data Availability

The datasets used and/or analysed during the current study are available from the corresponding author on reasonable request.

## Abbreviations

BC: Breast cancer
CTAB: Cetyltriethylammnonium bromide
DMSO: Dimethylsulfoxide
ESI: Electrospray ionization
GC-TOF-MS: Gas chromatography-time-of-flight-mass spectrometry
HRMAS-NMR: High-resolution magic angle spinning - nuclear magnetic resonance
IL-6: Interleukin-6
JAK2: Janus kinases 2
KEGG: Kyoto Encyclopedia of Genes and Genomes
N: Normal
NIH: National Institutes of Health
NMDS: Non-metric multidimensional scale
OPLS-DA: orthogonal partial least squares discriminant analysis
OTUs: Operational Taxonomic Units
PCA: Principal Component Analysis
PCoA: Principal Coordinate Analysis
PLS-DA: Partial least squares discriminant analysis
ROS: Reactive oxygen species
STAT3: Signal transducers and activators of transcription 3
TLR: Toll-like receptors
UHPLC: Ultra High Performance Liquid Chromatography System
