# Supplementary figures and images for: Alter between gut bacteria and blood metabolites and the anti-tumor effects of Faecalibacterium prausnitzii in breast cancer
Source: BMC Microbiol. 2020 Apr 9;20:82. doi: 10.1186/s12866-020-01739-1 (PMC7144064; doi:10.1186/s12866-020-01739-1)

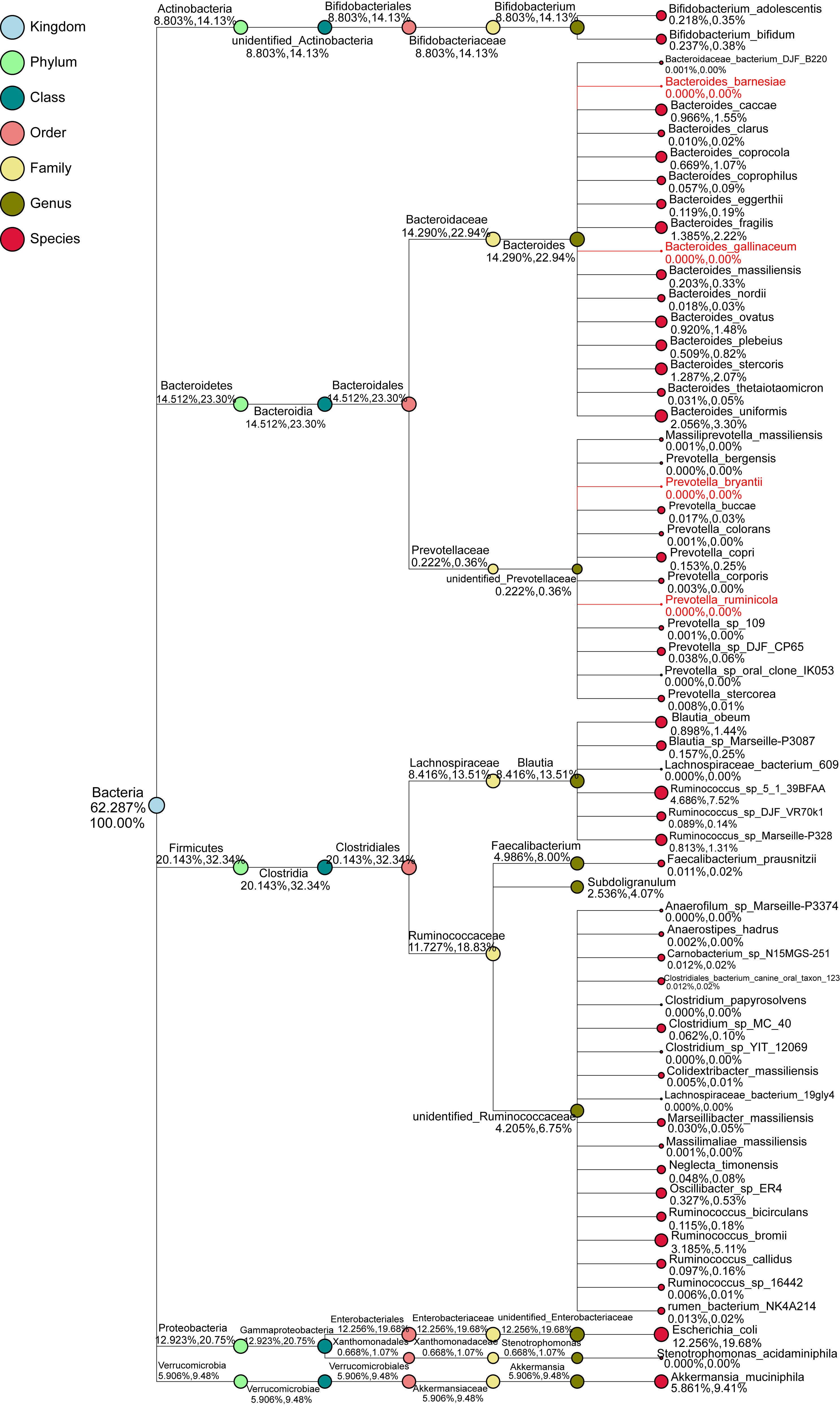

Supplement: Supplementary file 1 — Additional file 1: Figure S1 Species classification tree in a single sample. Circles of different colors indicate the level of classification. The size of the phase is abundance. The two numbers below the category name indicate relative percentages, the former accounting for the percentage of all species in the sample, and the latter representing the percentage of the sample selected. The classification of the red font indicates that the classification annotation does not exist in the sample, but is present in other analysis samples. [file 12866_2020_1739_MOESM1_ESM.bmp]

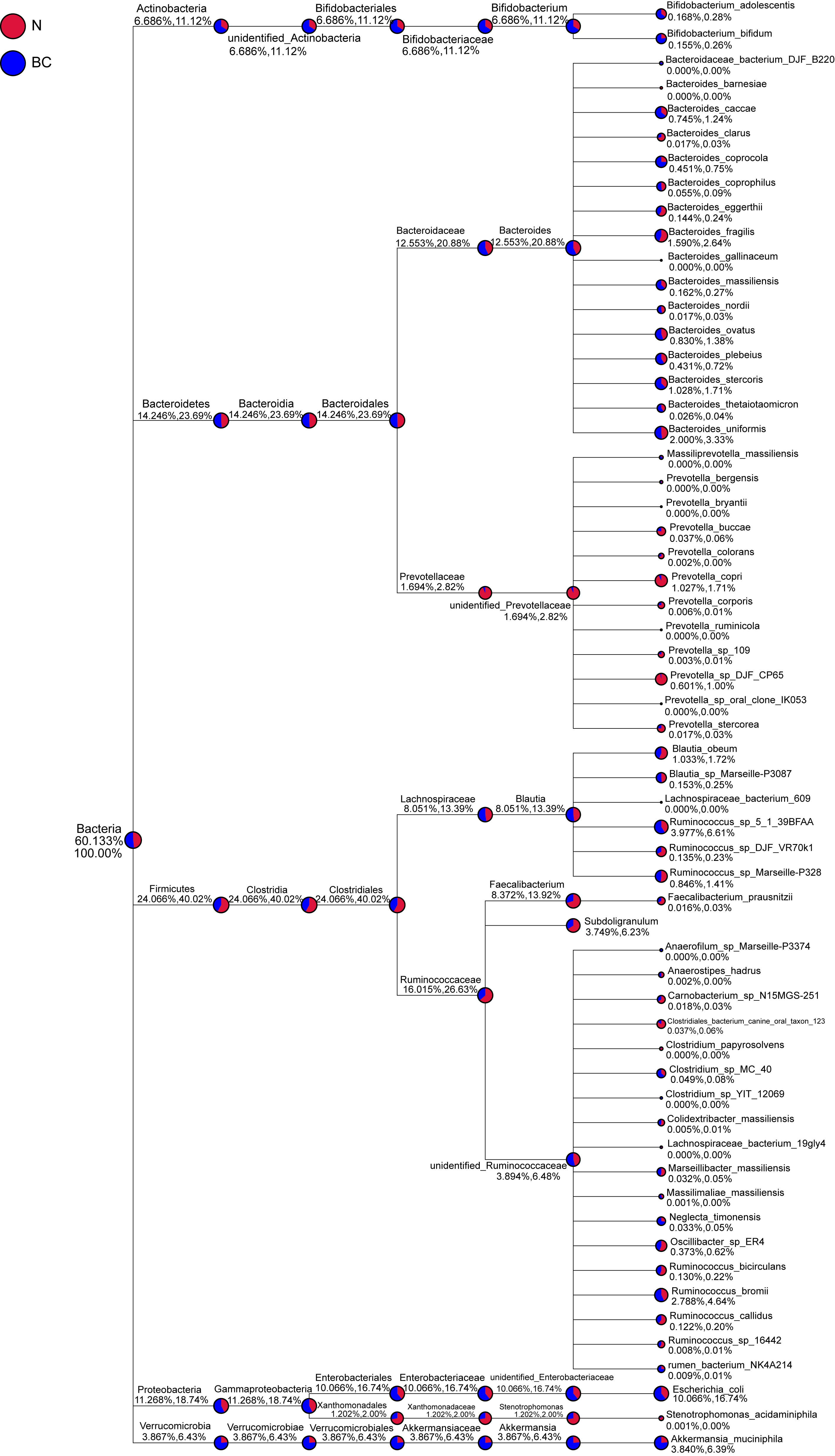

Supplement: Supplementary file 2 — Additional file 2: Figure S2 Species classification tree in grouped species. Fans of different colors in a circle represent different groups. The size of the sector indicates the proportion of the group’s relative abundance in the classification. The number below the category name indicates the average relative abundance percentage of all groups in the category. The former indicates the percentage of all species and the latter indicates the percentage of selected species. [file 12866_2020_1739_MOESM2_ESM.bmp]

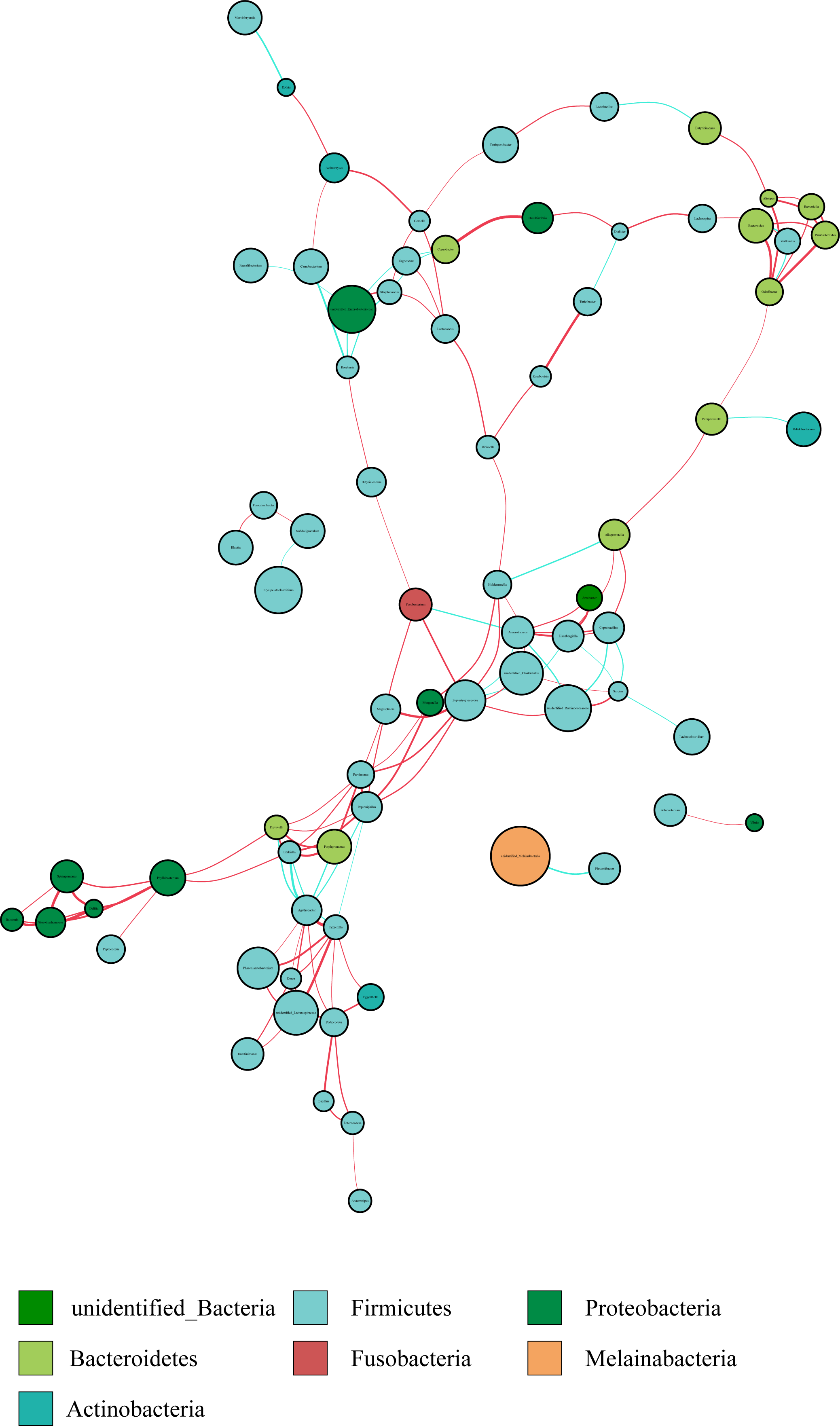

Supplement: Supplementary file 3 — Additional file 3: Figure S3 Network analysis. Different nodes represent genus, the node size represents the average relative abundance of the genus, and the nodes of the same gate have the same color. The line thickness between the nodes is positively correlated with the absolute value of the correlation coefficient of the species interaction, and the color and correlation of the line are positively and negatively correlated (red represent positive correlation, blue represent negative correlation). [file 12866_2020_1739_MOESM3_ESM.bmp]

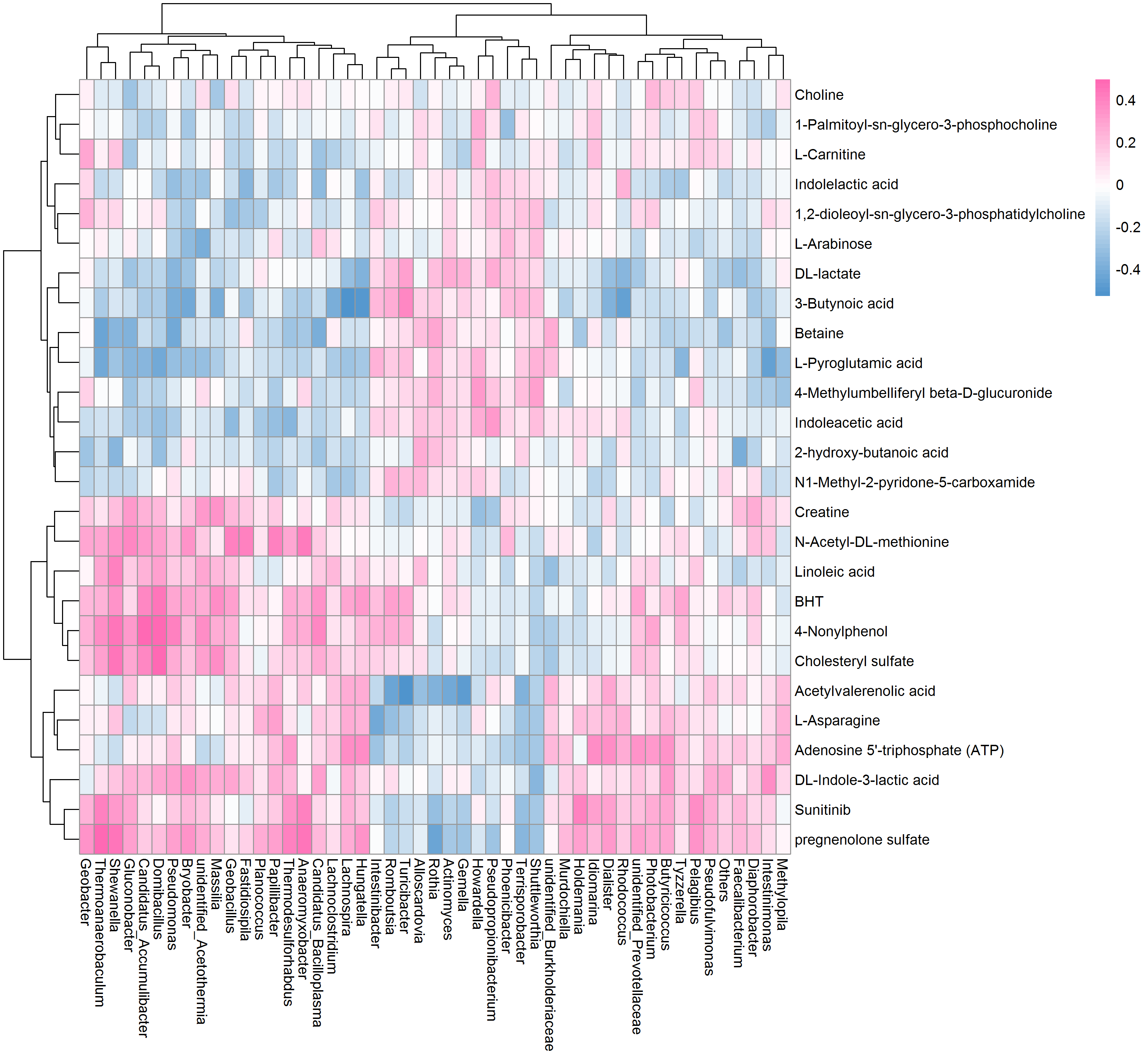

Supplement: Supplementary file 5 — Additional file 5: Figure S5 Cluster heat map of spearman correlation. The cluster heat map of spearman correlation hierarchical clustering analysis of significant differences in flora and metabolites [file 12866_2020_1739_MOESM5_ESM.bmp]

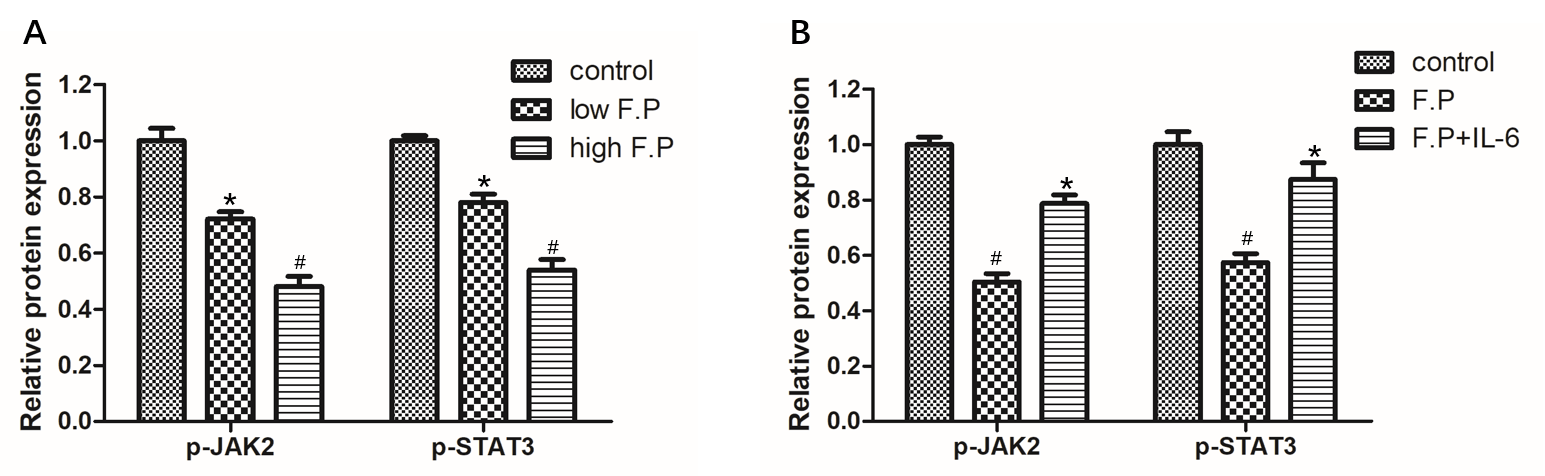

Supplement: Supplementary file 6 — Additional file 6: Figure S6 Quantitative analysis of protein expression. The protein band intensities were quantified with the Odyssey infrared imaging system. Data are presented as the mean ± standard deviation from three independent experiments. A. # indicates control vs high F.P group, P < 0.05; *indicates control vs low F.P group, P < 0.05. B. # indicates control vs F.P group, P < 0.05; *indicates control vs F.P + IL-6 group, P < 0.05. [file 12866_2020_1739_MOESM6_ESM.bmp]
